# Ca^2+^/calmodulin‐dependent protein kinase II and protein kinase G independently contribute to acutely enhance myocardial compliance with stretch

**DOI:** 10.14814/phy2.70709

**Published:** 2026-03-17

**Authors:** André M. Leite‐Moreira, João Almeida‐Coelho, Inês Falcão‐Pires, André P. Lourenço, Adelino F. Leite‐Moreira

**Affiliations:** ^1^ Cardiovascular R&D Centre ‐ UnIC@RISE, Department of Surgery and Physiology Faculty of Medicine of the University of Porto Porto Portugal; ^2^ Department of Anesthesiology São João Local Health Unit Porto Portugal; ^3^ Department of Cardiothoracic Surgery São João Local Health Unit Porto Portugal

**Keywords:** diastole, myocardial compliance, myocardial stiffness, protein kinases, stretch

## Abstract

The heart constantly adapts to acute hemodynamic load by increasing contractility and stretch‐induced compliance (SIC). The latter involves titin phosphorylation, notably by cGMP‐dependent protein kinase (PKG). Given that Ca^2+^/calmodulin‐dependent protein kinase II (CaMKII) also phosphorylates titin and is activated under acute stress via redox signaling, we hypothesized a role for CaMKII in SIC. We assessed passive tension (PT) decay in the 15 min following sudden stretch in isometrically contracting rabbit right ventricular papillary muscles, with or without PKG or CaMKII inhibition. Additionally, Wistar Han rat hearts were Langendorff‐perfused and acutely stretched or left unstretched. Skinned cardiomyocytes extracted from these hearts were analyzed for sarcomere length‐PT relationships before and after incubation with PKG, CaMKII, or both. Both kinases reduced PT, with diminished effects in pre‐stretched cells, suggesting prior kinase activation. PKG inhibition significantly blunted SIC, while CaMKII inhibition showed a similar but non‐significant trend. Our findings support that CaMKII contributes to SIC, likely via shared phosphorylation targets with PKG. These results provide mechanistic insight into SIC and suggest CaMKII as a potential modulator of diastolic function during acute stretch, such as in preload challenges.

## 1 INTRODUCTION

The heart continuously adapts to rapid changes in volume and pressure, which strain the tissues. The ensuing increases in systolic capacity are commonly known as the Frank‐Starling mechanism and the Anrep effect, respectively. More recently, acute stretch has also been shown to enhance myocardial diastolic compliance over the following minutes, allowing greater filling at lower end‐diastolic pressure. This novel adaptive biological mechanism, termed stretch‐induced compliance (SIC), was characterized as a sustained decrease in passive tension (PT) of over 40% that lasted for at least 15 min following an acute stretch. This response was consistently observed across multiple species and experimental preparations, including in isolated rabbit papillary muscles, intact rat hearts, and human myocardial strips.

Mechanistically, stretch‐induced compliance (SIC) is partly dependent on activation of cGMP‐dependent kinase (PKG) associated pathways and subsequent phosphorylation of the giant sarcomeric protein titin, the key determinant of myocardial passive tension (PT). The physiological importance of this adaptive mechanism is highlighted by its impairment in disease states, including left ventricular hypertrophy (LVH) (Leite‐Moreira et al., 2018) and acute myocardial ischemia (Leite‐Moreira et al., 2023).

The multifunctional Ca^2+^/calmodulin‐dependent protein kinase II (CaMKII), when chronically activated, is linked to cardiac hypertrophy, fibrosis, and arrhythmia (Beckendorf et al., 2018; Zhang, 2017). Yet, like PKG, it plays an important role in stretch, in the Anrep effect (Reil et al., 2020). Acute overload increases production of reactive oxygen species (ROS) by NADPH oxidase‐2 (Prosser et al., 2011), which can oxidize both PKG and CaMKII in specific residues and increase their substrate affinity and activity in an activator‐independent fashion (Erickson et al., 2008). Considering that CaMKII phosphorylates titin at the same region as PKG (Hamdani et al., 2013; Hidalgo et al., 2013), it is foreseeable that it may contribute to SIC.

We aimed to study the impact of CaMKII on SIC ex vivo and compare it with PKG.

## 2 METHODS

### 2.1 Isolated papillary muscles

Male New Zealand white rabbits (2–3 kg) were anesthetized with an intravenous administration of sodium pentobarbital (25 mg.kg^−1^). A left thoracotomy was performed, and beating hearts were quickly excised and immersed in modified Krebs–Ringer buffer (KRB; in mM: 98 NaCl, 4.7 KCl, 2.4 MgSO_4_, 1.2 KH_2_PO_4_, 4.5 glucose, 1.8 CaCl_2_, 17 NaHCO_3_, 15 sodium pyruvate, 5 CH_3_COONa, 0.02 atenolol, 30 2,3‐butanedione monoxime—BDM) at 30°C. pH was set at 7.4 with 95% O_2_/5% CO_2_. Papillary muscles were dissected from the right ventricle (RV), vertically mounted with the upper tendinous end attached to an electromagnetic length tension transducer (University of Antwerp, Belgium), and preload was initially set to 3 mN. After replacement with KRB without BDM, the preparations were stimulated at 0.2 Hz; the muscle length at which active force development was maximal was determined (L_max_), and the muscles were allowed to stabilize at 92% of L_max_. PT measurements were for 15 min of stretching from 92% of L_max_ to L_max_ after 20 min of incubation with either vehicle (*n* = 6), a CaMKII inhibitor (iCaMKII, Autocamtide 2‐related Inhibitory Peptide; Sigma‐Aldrich; 2 × 10^−5^ M; *n* = 6), a PKG inhibitor (iPKG, Rp‐8‐Br‐PET‐cGMPS; Sigma‐Aldrich; 10^−6^ M; *n* = 6), or both (*n* = 6).

### 2.2 Langendorff rat heart perfusion

Hearts from Wistar Han rats (250–300 g males, *n* = 12) were quickly excised via median sternotomy under anesthesia with sevoflurane 3% and placed in heparinized ice‐cold modified Krebs–Henseleit buffer (KHB; in mM: 115 NaCl−, 22 NaHCO_3_, 5.3 KCl, 1.4 CaCl_2_, 1.25 NaH_2_PO_4_, 12 glucose, 30 BDM) with pH set at 7.4 by gassing (95% O_2_/5% CO_2_). The aorta was immediately cannulated and perfused with 37°C KHB devoid of BDM or heparin at a constant pressure of 70 mmHg according to the Langendorff technique (Radnoti LLC, ADInstruments), as previously described (Leite‐Moreira et al., 2018). The hearts were apically vented for drainage and electrically paced above resting heart rate at 300 beats per minute. A plastic balloon was inserted in the left ventricle volume (LV) and volume was adjusted to reach a stable end‐diastolic pressure (EDP) of ≈3 mmHg (reference 0% volume). The balloon was then randomly inflated either to increase EDP to ≈12 mmHg (set as 100% volume) or left unaltered for the following 15 min (Stretched and Non‐stretched, respectively, *n* = 6 each). Upon completion, LV free‐wall samples were snap‐frozen in liquid nitrogen and stored at −80°C.

### 2.3 Isolated skinned cardiomyocytes

Myocardial samples (10–15 mg wet weight) from Stretched and Non‐stretched LV collected upon completion of isolated rat heart experiments were defrosted in cold relaxing solution, mechanically disrupted, and incubated for 5 mins in relaxing solution supplemented with 0.2% Triton X‐100 to remove membrane structures. Single cardiomyocytes were then attached with glue between a force transducer and an electromagnetic motor. Sarcomere length (SL)‐PT relationships, ranging between 1.8 and 2.3 μm at 0.1 μm‐step SL increases, were acquired in 3–4 mechanically isolated skinned cardiomyocytes, as reported (Gonçalves‐Rodrigues et al., 2020). SL‐PT relationship acquisition was carried out before and after incubation for 40 min with either purified CaMKIIδ (0.6 μg/mL; Merck Millipore) plus calmodulin (12.5 U/μL, Sigma‐Aldrich), purified PKG1α (1 U/mL; Sigma‐Aldrich) plus cGMP (10 μmol/L, Sigma‐Aldrich), or both kinases and activators. Force values were normalized to cardiomyocyte cross‐sectional area (kN.m^−2^).

### 2.4 Statistical methods

One‐way analysis of variance on ranks was employed to compare PT decay over time as percentage. SL‐PT relationships were compared using a repeated‐measures hierarchical linear model with condition (Stretched/Non‐stretched) and drug as fixed effects and subject as a random effect for a two‐tailed significance of 0.05.

## 3 RESULTS

**FIGURE 1:**
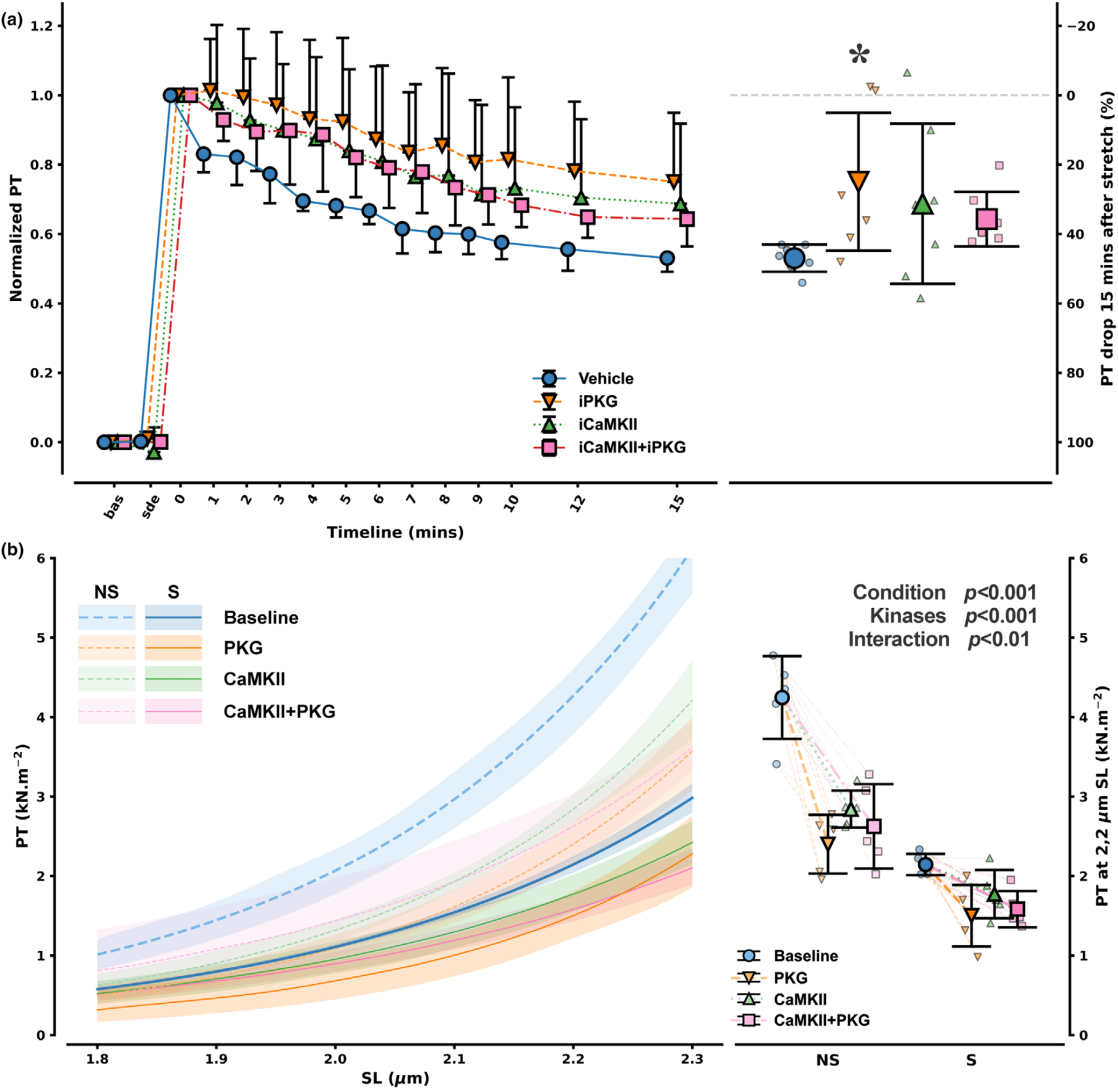
Modification of acute response to stretch in rabbit right ventricular papillary muscles (a) and rat skinned left ventricular cardiomyocytes (b) by modulation of protein kinase G (PKG) and Calcium/Calmodulin‐Dependent Protein Kinase II (CaMKII). In Panel a, upon baseline (bas) passive tension (PT) acquisitions at 92% of optimal sarcomere length (SL, L_max_), muscles (*n* = 7 per protocol) were incubated with vehicle (blue circles), PKG inhibitor (iPKG, orange down‐facing triangles), CaMKII inhibitor (iCaMKII, green upward‐facing triangles), or both (iCAMKII+iPKG, pink squares). After stable drug effect (sde), they were suddenly stretched to 100% of L_max_ and PT was sequentially recorded for 15 min. Representative tracings and errors (mean ± standard deviation) are shown on the left, whereas percent drop in PT from 100% L_max_ is shown on the right. In panel b, isolated myocytes from previously stretched hearts and their non‐stretched counterparts (Condition, S and NS represented in darker and lighter tones, respectively; *n* = 5 each) underwent length‐tension (SL‐PT) relationship at baseline (blue) and again after incubation with one of PKG (orange), CaMKII (green) or CaMKII+PKG (pink) (Kinases). For the sake of visual simplicity, 95% confidence intervals derived by exponential fits of the relationships fitting with 5000 bootstraps are presented for each intervention alongside point plots and error bars for PT at the SL of 2.2 μm. A minimum of 4 myocytes was averaged per animal. Baseline SL‐PT relationships were pooled for presentation.

Rabbit RV papillary muscles (Figure 1a), incubated with vehicle, demonstrated the characteristic SIC response. Following a sudden stretch from 92% to 100% L_max_, passive tension (PT) showed a clear, time‐dependent decay over the 15‐min observation period (47% ± 3.9%). We then assessed the role of PKG and CaMKII in this response using specific inhibitors, and all interventions attenuated the percentage of PT drop over 15 min. iPKG significantly (25% ± 20%) blunted the SIC response, attenuating the PT drop by approximately 50% compared to the vehicle (*p* = 0.018 with Dunn's test after Kruskal–Wallis). In contrast, iCaMKII alone did not result in a statistically significant change in the PT drop (31% ± 23%, *p* = 0.24). Similarly, the combined inhibition of both PKG and CaMKII (iCaMKII+iPKG) showed only a nonsignificant trend toward attenuation (36% ± 7.9%, *p* = 0.11).

In the isolated rat Langendorff heart preparations (Figure 1b), hearts subjected to an acute increase in balloon volume (Stretched group) showed a significant decrease in end‐diastolic pressure over 15 min, consistent with SIC (data not shown). Skinned cardiomyocytes were then isolated from these Stretched hearts and from Non‐stretched controls. Increased compliance, as denoted by a downward shift of the SL‐PT relationship, was observed in myocytes from stretched hearts (Figure 1b) compared with non‐stretched hearts (*p* < 0.001), highlighting the stretch‐induced increase in compliance as an intrinsic property of cardiomyocytes, and upon all incubations (*p* < 0.001).

In the Non‐Stretched cardiomyocytes, incubation with PKG, CaMKII, or both kinases resulted in a significant downward shift of the PT‐SL curve increasing compliance compared to their baseline. Nevertheless, the effect of PKG and/or CaMKII was less pronounced in myocytes from stretched hearts (*p* < 0.01), possibly suggesting previous phosphorylation of available sites by myocardial kinases during the stretch protocol.

## 4 DISCUSSION

Overall, our results provide new functional evidence that, in addition to the previously proposed PKG pathway, CaMKII contributes to enhancing myocardial compliance during acute stretch. In isolated papillary muscles, SIC response was, as expected, significantly blunted by PKG inhibition, while CaMKII inhibition alone did not reach statistical significance in this preparation. This could result from a small sample size, or it may reflect a more modest role for CaMKII compared to the PKG pathway in this particular setting. In skinned cardiomyocytes, incubation with CaMKII, similar to PKG, was sufficient to increase compliance, and this effect was blunted in myocytes from pre‐stretched hearts. The similarity in effect between both CaMKII and PKG and the lack of a synergistic effect on simultaneous inhibition or incubation might be explained by their shared phosphorylation targets on titin (Hamdani et al., 2013; Hidalgo et al., 2013).

Interestingly, despite sharing phosphorylation sites in titin N2B_us_ spring element with PKG, CaMKII also targets the PEVK region of titin, which, in turn, is associated with increased stiffness (Hamdani et al., 2013). Nevertheless, in our study we consistently observe overall decreased stiffness with CaMKII incubation in myocytes, which is in agreement with a previous study (Hidalgo et al., 2013), pointing to this as the dominant effect, at least in vitro.

The mechanism by which stretch activates CaMKII is still not fully elucidated. A recent study by Alim et al. showed that the afterload‐induced Anrep effect is mediated by NOS1‐dependent S‐nitrosylation of CaMKII at Cys290, which promotes autonomous CaMKII activity (Alim et al., 2022). Additionally, Reil et al. (2020) showed that the afterload‐induced increase in contractility was blunted in CaMKII‐knockout hearts, cementing CaMKII as a critical mediator of mechano‐chemo‐transduction. Our results complement these findings, suggesting that CaMKII is activated by stretch to modulate both systolic (Anrep effect) and diastolic (SIC) properties. A parallel activation pathway of CaMKII is through direct oxidation of its regulatory domain at Met 281/282 by ROS (Wang et al., 2021; Zhang, 2017). This ROS‐CaMKII link has been proposed as a fundamental evolutionary trade‐off, coupling ROS from physiological stress, such as exercise, to enhanced Ca^2+^ signaling and performance (Gömöri et al., 2022). Indeed, mechanical stretch from volume overload induces oxidative stress, leading to the oxidation of both CaMKII and PKG (Gömöri et al., 2022).

The pathological implications of this activation are context dependent. In disease states like type 2 diabetes, which are characterized by increased CaMKII activity, pharmacological inhibition of CaMKII has been shown to restore contractility and relaxation in isolated diabetic muscle. Notably, this restoration was found to be independent of changes in the Ca^2+^ transient, pointing to a direct myofilament‐level effect, which is consistent with our hypothesis of CaMKII acting on titin (Daniels et al., 2018). However, in chronic hypertrophy models, increased CaMKII oxidation and activity are ultimately associated with increased cardiomyocyte stiffness (Herwig et al., 2020). This suggests a potentially biphasic role for CaMKII: acute, physiological activation (as in SIC) is adaptive and increases compliance, but chronic, pathological activation in a high‐oxidative‐stress environment may contribute to diastolic dysfunction (Anderson, 2015; Beckendorf et al., 2018; Gömöri et al., 2022).

A primary limitation of this study is the lack of direct measurement of titin phosphorylation. In contrast to our previous work that first described SIC, where titin hyperphosphorylation upon stretch was measured using SYPRO Ruby and Pro‐Q Diamond staining, this could not be performed on the current samples. Therefore, our conclusions regarding kinase‐specific phosphorylation are based on circumstantial functional data, rather than direct phosphoprotein measurement. Furthermore, while redox‐signaling could be a potential mechanism for CaMKII activation during stretch (Erickson et al., 2008; Prosser et al., 2011), our experiments did not directly manipulate or measure ROS or redox conditions. On the other hand, we used two different mammalian models: rabbit RV papillary muscles and rat LV skinned cardiomyocytes. While interspecies differences in titin isoform expression and kinase regulation exist, the fundamental mechanisms of titin phosphorylation by these kinases are known to be highly conserved across mammals (Hamdani et al., 2013; Hidalgo et al., 2013). Although our results are consistent between two mammalian species, this study only focused ex vivo and in vitro preparations, limiting extrapolation to the in vivo setting.

## 5 CONCLUSION

In conclusion, this study provides new functional evidence supporting a role for CaMKII, alongside PKG, in the acute enhancement of myocardial compliance following stretch. Both kinases appear to independently contribute to this response, likely via shared phosphorylation targets on titin. Future studies, including direct phosphoprotein analysis, are warranted to further elucidate this mechanism in vivo and assess CaMKII's role in conditions where this adaptive response is impaired, such as in heart failure with preserved ejection fraction.

## FUNDING INFORMATION

This study was funded by national funds through the Portuguese Foundation for Science and Technology (FCT), under the scope of the Cardiovascular R&D Center—UnIC (UIDB/00051/2020 and UIDP/00051/2020) and national grant PTDC/DTP‐PIC/4104/2014.

## CONFLICT OF INTEREST STATEMENT

None to declare.

## ETHICS STATEMENT

Animal experiments were approved by the competent authorities (016531) and complied with the Guide for the Care and Use of Laboratory Animals (National Institutes of Health Publication no. 85–23, revised 2011) and the guidelines from Directive 2010/63/EU of the European Parliament on the protection of animals used for scientific purposes.

## Data Availability

The data that support the findings of this study are available from the corresponding author upon reasonable request.
